# *NPHS2* mutations account for only 15 % of nephrotic syndrome cases

**DOI:** 10.1186/s12881-015-0231-9

**Published:** 2015-09-29

**Authors:** Mara Sanches Guaragna, Anna Cristina GB Lutaif, Cristiane SC Piveta, Marcela L. Souza, Suéllen R. de Souza, Taciane B. Henriques, Andréa T. Maciel-Guerra, Vera MS Belangero, Gil Guerra-Junior, Maricilda P. De Mello

**Affiliations:** Centro de Biologia Molecular e Engenharia Genética, Universidade Estadual de Campinas, Campinas, São Paulo, Caixa Postal 6010 Brasil; Nefrologia Pediátrica, Departamento de Pediatria, Faculdade de Ciências Médicas, Universidade Estadual de Campinas, Campinas, São Paulo, Brasil; Centro de Investigação em Pediatria, Faculdade de Ciências Médicas, Universidade Estadual de Campinas, Campinas, São Paulo, Brasil; Departamento de Genética Médica, Faculdade de Ciências Médicas, Universidade Estadual de Campinas, Campinas, São Paulo, Brasil; Grupo Interdisciplinar de Estudos da Determinação e Diferenciação do Sexo, Faculdade de Ciências Médicas, Universidade Estadual de Campinas, Campinas, São Paulo, Brasil; Endocrinologia Pediátrica, Departamento de Pediatria, Faculdade de Ciências Médicas, Universidade Estadual de Campinas, Campinas, São Paulo, Brasil

**Keywords:** Steroid-resistant nephrotic syndrome, Children, Adolescents, Mutations, *NPHS2*, *NPHS1*, *WT1*

## Abstract

**Background:**

Nephrotic syndrome is traditionally classified on the basis of the response to standard steroid treatment. Mutations in more than 24 genes have been associated with nephrotic syndrome in children, although the great majority of steroid-resistant cases have been attributed to mutations in three main genes: *NPHS1*, *NPHS2* and *WT1*. The aims of this study were to identify mutations in these genes more frequently reported as mutated and to characterize each variation using different *in silico* prediction algorithms in order to understand their biological functions.

**Methods:**

We performed direct sequence analysis of exons 8 and 9 of *WT1*, 8 exons of *NPHS2* and 29 exons of *NPHS1*, including *NPHS2* and *NPHS1* intron–exon boundary sequences, as well as 700 bp of the 5′ UTR from both genes in 27 steroid-resistant patients aged between 3 months and 18 years.

**Results:**

Analysis of the *NPHS2* gene revealed four missense mutations, one frameshift mutation and three variations in the 5′ UTR. Four patients presented compound heterozygosis, and four other patients presented one heterozygous alteration only. *WT1* and *NPHS1* gene analysis did not reveal any mutations.

**Discussion:**

This is the first study focusing on genetics of SRNS in Brazilian children. Identification of mutations is important because it could influence physicians’ decision on patient treatment, as patients carrying mutations can be spared the side effects of immunosuppressive therapy and ultimately could be considered for kidney transplantation from a living donor.

**Conclusions:**

After molecular analysis of the genes more frequently reported as mutated in 27 steroid-resistant nephrotic syndrome patients, we identified *NPHS2* mutations confirming the hereditary character of the kidney disease in only 14.8 % of patients. Therefore, the next step is to perform a next generation sequencing based analysis of glomeluropathy-related panel of genes for the remaining patients in order to search for mutations in other genes related to steroid-resistant nephrotic syndrome.

**Electronic supplementary material:**

The online version of this article (doi:10.1186/s12881-015-0231-9) contains supplementary material, which is available to authorized users.

## Background

Nephrotic syndrome (NS) is considered as one of the main kidney diseases in children and is characterized by proteinuria, edema, hypoalbuminemia and hyperlipidemia [1]. Depending on the age at diagnosis, NS is usually classified as congenital (CNS) when it manifests *in utero* or during the first 3 months of life; as infantile when it occurs during the first year; as childhood when symptoms occur between 1 and 12 years of age; and as juvenile with onset between 12 and 18 years of age [2]. Approximately 90 % of patients show a favorable outcome after steroid treatment of NS and are termed steroid-sensitive NS (SSNS) patients. Clinically, however, these SSNS patients are not a homogeneous group, as some continue to develop frequent relapses (FRNS) or even follow a steroid-dependent course (SDNS) of the disease. The remaining 10 % are steroid-resistant NS (SRNS) patients who do not respond to steroids or to any other immunosuppressive therapy; they show most commonly histopathological findings of focal segmental glomerulosclerosis (FSGS) and generally progress to end-stage renal disease [2].

A single-gene cause is responsible for approximately 30 % of SRNS cases, as reported in a recent paper by Sadowski et al. [3] on the largest international cohort studied so far for monogenic causes of SRNS. By using an in-house developed high-throughput approach, the investigators examined 1783 families and identified mutations in 21 out of 27 genes encoding proteins mainly expressed in the glomerular filtration barrier (GFB). The clinical course in affected children differs considerably with respect to the mode of inheritance, age, renal biopsy results, response to immunosuppressive treatment and extrarenal manifestations. One of the main genes, *NPHS2* (OMIM *604766), encodes podocin, a member of the stomatin protein family that is localized at the slit membrane [4]. In the slit–diaphragm (SD) complex, podocin interacts with nephrin, a member of the immunoglobulin superfamily and the principal protein in the SD [5]. Podocin mediates the recruitment of nephrin into specialized microdomains of the cell plasma membrane where, in the so-called “lipid rafts”, both proteins play essential roles in the maintenance of the glomerular permeability barrier [6]. Moreover, podocin belongs to a family comprising more than 1800 proteins conserved throughout evolution that share approximately 150 amino acid domains similar to mitochondrial protein prohibitin (PHB). Many of the PHB domain proteins regulate membrane protein function by binding sterols and altering their local lipid environment [7]. Huber et al. [8] demonstrated that podocin binds to cholesterol and contributes to form a supercomplex of proteins and lipids that regulate ion channel complexes; the authors also showed that podocin interacts with the cytoplasmic tail of nephrin, thus potentiating the effect of nephrin cellular signaling.

Mutations in the *NPHS2* gene were originally described in families with autosomal recessive SRNS (nephrotic syndrome type 2, OMIM #600995) [8] but later were found to be present also in sporadic cases [9], occurring in approximately 40 % of familial cases and 6–17 % of sporadic SRNS cases [9–11]. Familial and sporadic cases with compound heterozygous or homozygous mutations in *NPHS2* typically have disease onset between 3 months and 5 years of age. Recently, Bouchireb et al. [12] pointed out that not all ethnic groups share the same frequency of mutations in this gene, following an extensive review of *NPHS2* mutations in SRNS patients between 1999 and 2013. It was found that African-American and Asian patients show a low prevalence of *NPHS2* mutations.

Regarding the *NPHS1* gene (OMIM *602716) that encodes nephrin, two major mutations were originally identified in Finnish patients with CNS of the Finnish type (OMIM #256300) [13]. In non-Finnish patients, a large number of different mutations have been described [13–15]. In addition to the typical features of CNS, mutations in nephrin were also associated with childhood-onset SRNS in patients aged from 5 months to 8 years [16]. Furthermore, Koziell et al. [17] described the coexistence of mutations in *NPHS2* and *NPHS1* genes in patients with milder CNS.

A third important gene involved in the genetics of NS is *WT1* (OMIM *607102), which encodes a zinc finger transcription factor. *WT1*-dominant mutations were originally identified to be responsible for Wilms’ tumor (WT), a childhood kidney cancer (OMIM #194070) [18, 19, 20]. *WT1* mutations were also associated with Denys-Drash syndrome (OMIM #194080) [21, 22] and Frasier syndrome (OMIM #136680) [22]. However, dominant *de novo* mutations in exons 8 and 9 have been found as the cause of approximately 2.7–7 % of sporadic cases of isolated SRNS in infants and children. In a recent study, Lipska et al. [23] used the PodoNet cohort to describe the genotypic and phenotypic spectrum of *WT1*-associated kidney disease and found *WT1* mutations in 6 % of sporadic SRNS patients, confirming the prevalence found by previous smaller studies.

Molecular data are clinically relevant to SRNS patients, because children who do not respond to corticosteroids and present with mutations in any of these genes can be spared the adverse effects of corticosteroid treatment. In addition, patients with mutations have a reduced risk of developing FSGS after renal transplantation, compared to patients without mutations, at a rate of 8 % versus 30 %, respectively [11].

In our study, we performed sequence analysis of the entire *NPHS2* and *NPHS1* genes and exons 8 and 9 of the *WT1* gene in 27 SRNS Brazilian patients, including seven unrelated familial cases and 20 sporadic cases. We present nucleotide changes found after screening these 27 SRNS patients.

## Methods

### Patients

Between 2008 and 2013, 27 SRNS patients, comprising seven unrelated familial cases and 20 sporadic (isolated) cases, were included in this study. Prerequisites for enrollment included the presence of NS for at least 1 year and the age at disease onset being between 3 months and 18 years of age. The median age at disease onset was 4 years. From the 27 patients, 15 were males and 12 were females. Resistance to steroids was defined by nephrologists who followed up the patients, according to the International Study of Kidney Disease in Children definition [24]. The definition of chronic kidney disease (CKD) was classified according to the National Kidney Foundation for patients aged over 2 years [25]. Eight of 27 patients progressed to CKD and had already had a kidney transplant. Table 1 presents the age at disease onset, age at CKD diagnosis and at transplantation, and biopsy and molecular findings. The Ethics Committee from Faculdade de Ciências Médicas at Universidade Estadual de Campinas approved this study. Written informed consent for the study was obtained from all patients and their parents.

**Table 1:** Clinical data of SRNS patients with *NPHS2* and *NPHS1* alterations

Patient number	Gender	Sporadic/ familial^a^	Age at onset (years)	Renal Biopsy^b^	Age of onset to CKD - V (Yes/No)^c^	Age of transplant (Yes/No)	*NPHS2*		
							Nucleotide change	Effect on coding	Ref.
					(years)	(years)			
Steroid Resistant cases with two NPHS2 heterozygous alterations									
P6	F	spo	12	FSGS	Yes/15	Yes/15	c.686G > A	p.Arg229Gln	[37]
							c.851C > T	p.Ala284Val	[10]
P67	F	spo	2.2	DMP	No	No	c.686G > A	p.Arg229Gln	[37]
							c.928G > A	p.Glu310Lys	[38]
P103	F	fam	1.2	FSGS	No	No	c.714delG	p.Lis239Argfs*13	ps
							c.779 T > A	p.Val260Glu	[12]
P154	M	fam	13	FSGS	No	No	c.686G > A	p.Arg229Gln	[37]
							c.851C > T	p.Ala284Val	[10]
Steroid Resistant cases with one NPHS2 heterozygous alteration									
P111	F	spo	3	FSGS	No	No	c.-164C > T	-	ps
P68	M	spo	4	CO	No	No	c.-196C > G	-	ps
P85	M	spo	6.11	CO	Yes/8	Yes	c.-537_-531delCTTTTTT	-	[40]
P72	M	fam	16	FSGS	Yes/18	Yes/18	c.686G > A	p.Arg229Gln	[37]

^a^ *Spo*: sporadic; *Fam*: familial;^b^ *FSGS*:focal segmental glomerular sclerosis; *DMP*: diffuse mesangial proliferation; *CO*: complex = minimal change disease or diffuse mesangial proliferation or focal segmental glomerular sclerosis; *CKD*: ^c^chronic kidney disease; *n.a*.: not analyzed; *nd*: not determined

### Molecular analysis of *NPHS2*, *NPHS1* and *WT1* genes

Genomic DNA was isolated from blood samples using standard methods [26]. Amplification of all coding exons, exon–intron boundaries and 700 bp of the 5′ UTR end from both *NPHS2* and *NPHS1* genes were performed by polymerase chain reaction (PCR). For *WT1* gene we performed amplification of exons 8 and 9 only (for specific primers, see in Additional file 1: Tables S1, S2 and S3). After PCR reactions and direct sequencing of products (see Additional file 1), Chromas Pro v.1.5 and CLC Sequence Viewer v.6.6.2, were used to analyze and compare these sequences with the *NPHS2*, *NPHS1* and *WT1* reference sequences in the Ensembl database (ENSG00000116218, ENSG00000161270, ENSG00000184937, respectively; http://www.ensembl.org/index.html).

### Bioinformatics analysis

The impact of missense and 5′ UTR variations on podocin or nephrin structure and function was evaluated using selected bioinformatics tools. SIFT (***S****orting****I****ntolerant****F****rom****T****olerant*; http://sift.jcvi.org/) [27], Polyphen (***Poly****morphism****Phen****otyping*; http://genetics.bwh.harvard.edu/pph2/) [28] and Align GVGD (http://agvgd.iarc.fr/agvgd_input.php/) [29] were used to assess potentially damaging effects of non-synonymous amino acid substitutions (see in Additional file 1: Table S4). In order to verify conserved amino acid residues, we performed multiple alignment analysis using ClustalW program [30] that lines up orthologs from different species to reveal the identities, similarities and differences. Human nephrin and podocin sequences were compared with orthologous sequences available in UniProt data bank (http://www.uniprot.org/). Moreover, in order to better understand if missense mutations affected exonic splicing enhancers or silencers (ESEs and ESS, respectively) leading to splicing defects, we further performed prediction analysis using two premRNA splicing prediction tools: Human Splicing Finder (HSF) [31] and SpliceAid2 [32]. The three 5′ UTR heterozygous alterations were analyzed using Alibaba2 gene regulation program (http://www.gene-regulation.com/pub/programs/alibaba2/index.html) that predicts transcription factor binding sites in the DNA query using data from TRANSFAC® Public eukaryotic transcription factors [33].

## Results and discussion

Since the discovery that inherited structural defects in the GFB are responsible for a large proportion of SRNS cases, major breakthrough studies on the discovery of genes and their implication in the disease have been carried out mainly in European and North American countries [3, 4, 14, 34, 35, 36]. In Brazil, genetic screening of patients with NS in childhood is still incipient. This is the first study focusing on genetics of SRNS in Brazilian children. Identification of mutations is important because it could influence physicians’ decision on patient treatment, as patients carrying mutations can be spared the side effects of immunosuppressive therapy and ultimately could be considered for kidney transplantation from a living donor.

Here we present results for a cohort of 27 SRNS patients from a public hospital that is a reference center in the South East region of Brazil where approximately 200 patients with NS are routinely followed up. Of note, the 27 SRNS patients represented a frequency of 13.5 % (27/200), which is in line with the reported worldwide frequency of 10–20 % for this form of the disease [2, 11]. However, the cohort was not representative of the entire country, since the Brazilian population consists of about 205 million of highly miscegenated people with diverse and regional features. However, there is no other genetic study on NS patients in Brazil for comparison.

Only four patients (two familial and two sporadic cases) representing a total of 14.8 % showed two heterozygous alterations that confirmed the hereditary character of their kidney disease. Mutations in *WT1* exons 8 and 9 and *NPHS1* gene were not found. *NPHS2* analysis, however, showed eight alterations: four missense mutations, one frameshift mutation and three variations in the 5’ UTR (Table 1).

### Sporadic cases

**Fig. 1:**
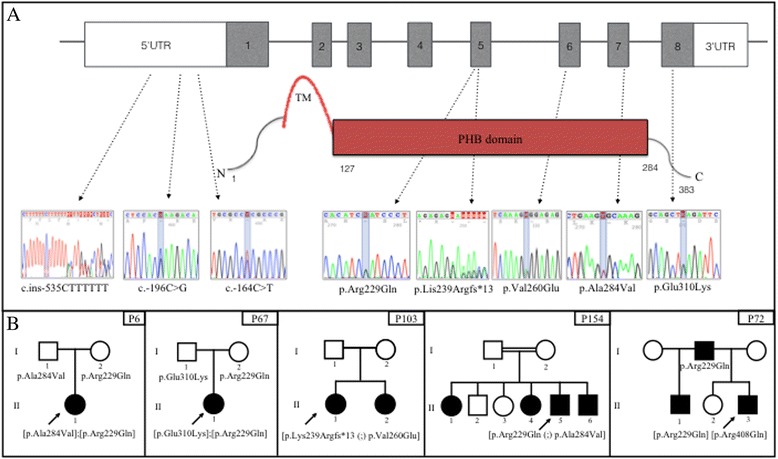
Schematic structure of *NPHS2*/podocin showing the position of alterations and patient’s heredograms. **a** Eight exons of *NPHS2* gene and podocin illustration showing electropherograms of the three 5’ UTR variants, four missense mutations and the frameshift mutation in the corresponding exons/domains identified in five SRNS patients. TM = transmembrane domain; PHB = prohibitin domain. **b** Sporadic cases P6, P67 (*first two boxes on the left*) and family cases P103, P154 and P72 pedigrees. Black squares and circles represent patients with kidney disease. Arrow indicates the index case. Mutations are annotated below the individuals that had their *NPHS2* or *NPHS1* genes screened. P103 (II1) and her sister (II2) presented [p.Lys239Argfs*13 (;) p.Val260Glu] association. P154 (II4) and her two brothers (II5 and II6) presented the [p.Arg229Gln (;) p.Ala284Val] association

**Fig. 2:**
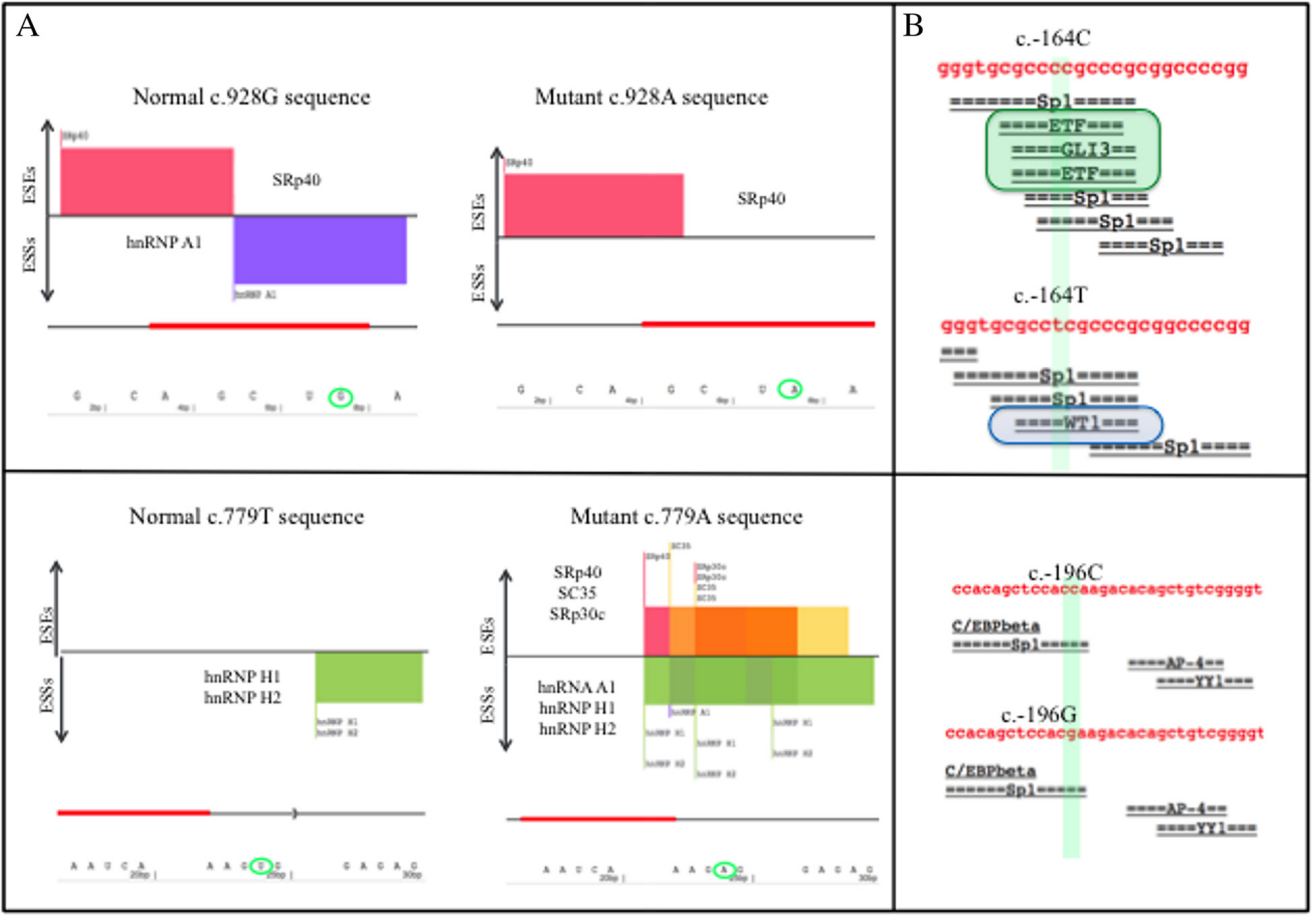
Splice Aid2 and Alibaba2 gene regulation *in silico* predictions. **a** Splice Aid2 graphic outcomes from c.928G > A (p.Glu310Lys) and c.779 T > A, (p.Val260Glu) substituions. Top: For c.928G > A the ESS recognition site for hnRNP A1 is broken after G > A substituion. Below: after c.779 T > A substitution (*right*) the histogram result shows that, besides the two hnRNPs (H1 and H2) proteins that putatively binds the normal ESS recognition site, a new motif is created, with the potential of being recognized by the hnRNP A1 and by other four SR proteins. Arrows on the left of histograms indicate proteins that bind ESEs and ESSs, respectively. The bars have variable width and height respectively related to the number of nucleotides of the binding site and to its score (binding affinity). Next to each bar there is the label showing the name of the protein predicted to bind to the recognition motif. Below the histogram there are two lines: one with red bars, representing the recognition motifs and with a bracket representing a putative splicing site; the other line represents the DNA sequence analyzed. A green circle highlights the nucleotide substitution. **b** Alibaba2 gene regulation prediction analysis showing above: wild c.-164C and mutant c.-164 T sequences (*highlighted*). Wild sequence was recognized by putative transcription factors ETF-1 and GLI3 and, after substitution, these sites were abolished and a new recognition site for putative WT1 transcription factor was created. Below: no change after c.-196C > G substitution

Among the 20 sporadic cases, the c.686G > A transition in exon 5 leading to p.Arg229Gln [36] was identified in association with two different pathogenic missense mutations (Table 1). Patient P6 was compound heterozygous [p.Arg229Gln];[p.Ala284Val], as indicated by the c.851C > T nucleotide transition [9] observed from *NPHS2* sequencing. Compound heterozygosis for the two mutations was demonstrated by the analysis of patient P6’s father carrying p.Ala284Val and mother carrying p.Arg229Gln (Fig. 1). Patient P67 was compound heterozygous [p.Arg229Gln];[p.Glu310Lys] which resulted from c.928G > A transition in exon 8 [37]. Individual mutations were also inherited from each parent, indicating an autosomal recessive inheritance for SRNS in both cases (Fig. 1). The p.Arg229Gln variant is the most frequently reported non-synonymous *NPHS2* variant in Caucasians; there are reports on its allele frequency showing that it is more frequent among European descendants than African descendants in the United States [10, 11, 37]. Tsukaguchi et al. demonstrated that trans-association of p.Arg229Gln with pathogenic mutations may result in a less severe phenotype such as late-onset SRNS. The most commonly associated mutation is p.Ala284Val, and notably this combination is predominant among South American populations from Chile and Argentina with adult-onset SRNS [37]. Recently, Tory et al., observed that p.Arg229Gln podocin presented a subcellular mislocalization only when co-expressed with podocins carrying amino acid substitutions encoded on exons 7–8, but not with substitutions encoded on exons 1–6 [38]. The authors suggested that disease-associated 3′ mutations exert a dominant-negative effect on p.Arg229Gln podocin, on the basis of a structural model of dimerization; heterodimers of p.Ala284Val podocin with p.Arg229Gln podocin showed an altered three-dimensional structural arrangement, and the authors therefore proposed this different structure contributed to the retention of p.Arg229Gln podocin within cytoplasmic compartments. However, when pathogenic mutations were associated with wild-type podocin, they behaved as recessive alleles. In summary, Tory et al. [39] described a phenomenon in an autosomal recessive disorder, in which the pathogenicity of one allele depended on that of the other allele, and they proposed a pattern of mutation-dependent recessive inheritance in *NPHS2*-associated SRNS that is beyond Mendel’s laws. Based on these criteria, the pathogenic association [p.Arg229Gln]; [mutEx7–8] was identified in both patients P6 and P67. Patient P6 was diagnosed with SRNS when proteinuria occurred at the age of 12 years, thus characterizing a late-onset form of the disease. Renal biopsy revealed FSGS, and the patient progressed to CKD in 3 years when she had a kidney transplant; after approximately 1 year post-transplant, her renal function has now returned to normal. In contrast, patient P67 developed proteinuria at 2 years and 2 months of age, and renal biopsy demonstrated diffuse mesangial proliferation (Table 1). The same [p.Arg229Gln];[p.Glu310Lys] association was identified by Machuca et al. [37] in three patients, but all three presented with FSGS. *In silico* evaluation using SpliceAid2 and HSF splicing prediction tools for c.686G > A (p.Arg229Gln), c.851C > T (p.Ala284Val) and c.928G > A (p.Glu310Lys) gave the following results: SpliceAid2 prediction indicated that c.686A did not show significant modifications in splicing motifs; c.851 T did not modify protein-binding recognition motifs; however, c.928A variant disrupted a hnRNP A1 protein-binding motif in the mutated sequence (Fig. 2). HSF prediction indicated that c.686A did not show modification in sequence motifs (data not shown); c.851 T created an exonic ESS site; for c.928A substitution, there was an ESE site broken after HSF prediction (Table 2).

**Table 2:** Exonic mutations *in silico* prediction

Enhancer motifs analysis through ESE finder matrices for SRp40, SC35, SF2/ASF and SRp55 proteins							
Sequence Variant	Sequence position^a^	Linked SR^b^ protein	Reference motif	Linked SR^b^ protein	Mutant motif		Variation
			(value 0–100)^c^		(value 0–100)^c^		
c.779 T > A	776	-	-	SF2/ASF	AAGAGGA (80.37)		New ESS site
c.851C > T	845	-	-	SF2/ASF	CTGAAGT (75.38)		New ESS site
c.928G > A	923	SF2/ASF	CAGCTGA (78.10)	-	-		Site broken
Potencial splice sites prediction through HSF matrices							
Sequence Variant	Sequence position	Splice site type	Motif	New splice site	Wild type^d^	Mutant^d^	Variaton
c.779 T > A	769	Acceptor	GGAATCAAAGTGGA	ggaatcaaagagGA	38.61	67.56	New site +74.98

^a^Position in cDNA from ATG codon; ^b^SR = serine/arginine rich proteins; ^c^ = Consensus values go from 0 to 100 and the threshold is defined differently for each algorithm. Threshold for SF2/ASF from ESE finder matrices is 72.98. Every signal with a score above the defined threshold is considered to be a potential ESE/ESS. ^d^For HSF matrices consensus values go from 0 to 100 and threshold is 65. Every signal with a score above the threshold is considered to be a splice site (donor or acceptor). When a mutation occurs if the WT score is under the threshold and the score variation is above +10 % for HSF it is considered that the mutation creates a new splice site

Considering compound heterozygosis for the two mutations, the percentage of patients with sporadic SRNS was 10 % (2/20). This result is in accordance with data published by others that indicated frequencies for patients bearing two heterozygous mutations varying from 6 to 17 % among sporadic SRNS cases [9–11].

Moreover, among the sporadic cases, there were also three cases in which we identified only one heterozygous alteration, the promoter variants c.-164C > T, c.-196C > G and c.-537_-531delCTTTTTT in patients P111, P68 and P85, respectively. Di Duca et al. [39], who studied regulatory elements in the *NPHS2* promoter, described c.-537_-531delCTTTTTT (rs146791300, MAF = 0.069; http://www.ncbi.nlm.nih.gov/) variant as a “functional polymorphism” that downregulated the gene expression of podocin by 85 % when transfected in podocytes. As there are no records of c.-164C > T and c.-196C > G variants in the database (http://www.ensembl.org), we screened the 700 bp of the 5′ UTR in 278 control subjects. We identified the c.-196G in heterozygosis in two controls (2/278, <1 %), but neither c.-537_-531delCTTTTTT nor c.-164 T were found, suggesting a possible role as disease-causing mutations. Alibaba2 gene regulation prediction algorithm showed that the C > G transversion in position c.-196 did not disrupt the SP1 transcription binding site located in this region (data not shown). However, this was not the case for C > T transition in −164 position that created a new WT1 transcription factor binding site (Fig. 2). It is well known that WT1 plays a key role during kidney and genital development [19, 20]. However, after birth, WT1 expression persists in the glomerular visceral epithelial cells where the protein probably contributes to the functional maintenance of differentiated podocytes [40]. In fact, it was previously demonstrated that WT1 binds and activates a glomerular-specific *NPHS1* enhancer *in vitro* [41]. Although there are no reports of WT1 acting as a trans-regulator element of the *NPHS2* promoter, we cannot discard a possible role for WT1 in this process, since mechanisms by which transcription factors regulate the expression of their target genes in podocytes are not yet fully understood. Recently, He et al. [42] identified a putative enhancer at 743 bp in the human *NPHS2* promoter that is combinatorially regulated by Lmx1b and FoxC transcription factors in podocytes. Therefore, with respect to c.-164C > T identified in our study, further expression and electrophoretic mobility shift assays are essential to better understand its role in podocin production. What is already known is that, in proteinuric renal disease, podocin expression is a dynamic process in which dramatic decreases or abnormal distributions of podocin expression in glomeruli of children with NS have already been demonstrated [43].

### Familial cases

When considering only familial cases, two out of seven unrelated cases presented two heterozygous alterations. The new frameshift p.Lys239Argfs*13 was identified in association with the already described p.Val260Glu missense in patient P103 and her sister (Fig. 1). We considered compound heterozygosis as the most probable genotype characterizing an autosomal recessive inheritance in this family, although we could not analyze their parent’s *NPHS2* gene. The frameshift p.Lys239Argfs*13 results from the c.714delG deletion in exon 5, and putatively produces amino acid changes from residues 239 to 251 and creates a premature stop codon at residue 252. Therefore, this mutation might affect podocin interaction with nephrin via the PHB domain, since podocin PHB domain comprises amino acids 127 to 284 [44]. The missense p.Val260Glu is the result of c.779 T > A transversion in exon 6 [11]. The protein resulting from this substitution is retained in the endoplasmic reticulum and loses its ability to recruit nephrin in lipid rafts [45]. After *in silico* evaluation of c.779 T > A (p.Val260Glu) by HSF and SpliceAid2 splicing prediction tools, this substitution was classified with the potential of interfering with splicing in different ways: by activating a new exonic cryptic acceptor splice site, as predicted by HSF matrices; and by creating an ESS site, as predicted by exonic regulatory sequences (ESRs) algorithm [46] as well as HSF matrices (Table 2). SpliceAid2 prediction indicated that, after T > A substitution, new ESE and ESS putative binding motifs were created that might be recognized by different serine and arginine-rich (SR) proteins and heterogeneous nuclear ribonucleoproteins (hnRNP), respectively (Fig. 2). In patient P154, we also identified the [p.Arg229Gln (;) p.Ala284Val] association, as described for patient P6. Patient P154 is a 13-year-old boy with FSGS. He has five siblings, and we analyzed the *NPHS2* gene in two of the siblings, a sister and a brother with FSGS who also presented the [p.Arg229Gln (;) p.Ala284Val] association. Analysis of the mother revealed p.Arg229Gln; however, a DNA sample from the father was not available for analysis. Our findings also included an SRNS patient with only one heterozygous alteration in the *NPHS2* gene, which diverged from the concept that SRNS is an autosomal recessive disease. The p.Arg229Gln substitution was identified in patient P72 whose father also showed the disease-associated polymorphism. Patient P72 had the disease onset at 16 years of age when he was diagnosed with FSGS upon renal biopsy. He progressed to CKD and had kidney transplant at the age of 18 years. His father was diagnosed with FSGS and had kidney transplant about 30 years ago, with no FSGS recurrence thereafter. The family also reported that the patient’s brother died of renal failure. Therefore, this family history suggests a possible genetic origin for the disease. Since *NPHS2* mutations are expected to act in a recessive mode in SRNS patients, our hypothesis is that mutations in other genes might be contributing to the disease in this family. *NPHS1* analysis revealed the c.1223G > A substitution in exon 10, leading to the p.Arg408Gln variant [14] (Fig. 1). There is some evidence, however, that this non-synonymous substitution is probably neutral, since it has been described as a polymorphism frequently found in homozygosis and heterozygosis in control individuals [15, 47]. In order to verify its frequency among Brazilians, we analyzed 100 controls and found 2 % were heterozygous for this variant (data not shown). In addition, patient P72’s father did not show this *NPHS1* variant, which led to the conclusion that there might be a mutation in one of the many other genes implicated in NS that were not screened in the present work. Besides these three cases with mutations, the other four cases with a family history of SRNS did not show any mutations in the *NPHS2* gene. Therefore, the percentage of familial cases carrying two *NPHS2* mutations in our study was 28.57 % (2/7), whereas other groups reported percentages of around 40 % [9–11]. It is likely the small study sample size here could explain this difference in our frequency result.

Furthermore, we also found that, among four patients bearing two heterozygous mutations, two (2/4, 50 %) presented the [p.Arg229Gln];[p.Ala284Val] association already described as predominant in South American populations [37]; apparently, this is also a common association in South Eastern Brazil. Patient P6’s grandparents are from Brazil, and the patient herself does not have a “hispanic” background. She was the only patient who had available information about her grandparents’ origin. The data reported here also demonstrated a considerable incidence of single heterozygous *NPHS2* alterations, but we cannot correlate them with a favorable outcome, since two patients (P85 and P72) progressed to CKD Class V (Table 1). Of note, one of these two patients presented a promoter variation, and, in fact, we identified three promoter variations that might downregulate podocin in the GFB of these patients. Therefore, further functional studies are necessary, in order to evaluate if these promoter variants alter the regulatory mechanisms and influence the maintenance of the barrier’s functional integrity.

## Conclusions

Our study reports the identification of *NPHS2* mutations in only 14.8 % of both sporadic and familial SRNS cases in the Brazilian patients analyzed, after screening for mutations in the *NPHS2*, *NPHS1* and *WT1* genes. The study did not reveal mutations in *NPHS1* and *WT1*. Therefore, target-oriented next generation sequencing analysis of glomerulopathy-related panel of genes is recommended to search for mutations in other genes related to SRNS.

## Additional file

Additional file 1:
**Supplemental Information.** **Table S1.** *NPHS2* specific primers. **Table S2.** *WT1* exons 8 and 9 especific primers. **Table S3.** *NPHS1* specific primers. Methods (PCR reactions and sequencing). **Table S4.** *NPHS2* and *NPHS1* missenses *in silico* predictions. (DOC 84 kb)
