# Supplementary material for: NPHS2 mutations account for only 15 % of nephrotic syndrome cases
Source: BMC Med Genet. 2015 Sep 29;16:88. doi: 10.1186/s12881-015-0231-9 (PMC4589073; doi:10.1186/s12881-015-0231-9)
Supplement: Additional file 1: — Supplemental Information. Table S1. NPHS2 specific primers. Table S2. WT1 exons 8 and 9 especific primers. Table S3. NPHS1 specific primers. Methods (PCR reactions and sequencing). Table S4. NPHS2 and NPHS1 missenses in silico predictions. (DOC 84 kb) [file 12881_2015_231_MOESM1_ESM.doc]

**Supplemental Information**

**Table S1: *NPHS2* specific primers**

| **Fragment** | ***Primer forward***  **5` 🡺 3`** | ***Primer reverse***  **5` 🡺 3`** |
| --- | --- | --- |
| **5’s-1.2as** | GTAGGGAGGAGAGAAAGGCATC | TGCCCTCTTGTTCTCCTTGTG |
| **1s-1as** | GGGCGCAGTCCACAGCTC | GGCCCCGAGACCAGTATATAGTG |
| **2s-2as** | GCCCTGTGAACTCTGACTACTCTG | GGAGCACCAGGAAGGGAATG |
| **3s-3as** | ATCAAAATTCTGTCTATGGGTTC | TGGCATGTGGGCTCTCTG |
| **4s-4as** | CCATGACTAAAAGGACCACACAG | CATTCCCTAGATTGCCTTTGC |
| **5s-5as** | TTAAATAAAGGGTAGGCCAACTCC | CCTAAGGGATGGAACTGGCC |
| **6s-6as** | CCTCTTGGGGTAACATTCACAG | TGCGCCTGGCCTAAAATG |
| **7s-7as** | CAAAACCTGCTGTGCTGATAATG | GAGGGATTGATGTGTGTGGAGG |
| **8s-8.1as** | TTCTATGCTTAACCGTGCTTGC | GCTGTTTCCCATAATTGCTCTG^a^ |
| **8.1s-8as** | ATGTTATAGGAAGGATGGGGC^a^ | CTCCCTCAGATTTTAAGCCAC |

^a^Primers specific for sequencing.

**Table S2: *WT1* exons 8 and 9 especific primers**

| **Fragmento** | ***Primer sense***  **5’ 🡺 3’** | ***Primer* *antissense***  **5’ 🡺 3’** |
| --- | --- | --- |
| **8s-8as** | TACCCTAACAAGCTCCAGCG | TCTCTCAACTGAGTCTAAACCTTAG |
| **9s-9as** | TGAGGCAGATGCAGACATTG | GAGAATCATGAAATCAACCCTAG |

**Table S3: *NPHS1*** specific primers

| **Fragment** | ***Primer forward***  **5’ 🡺 3’** | ***Primer reverse***  **5’ 🡺 3’** |
| --- | --- | --- |
| **5`s – 1.1as** | CCTGGCCTGCTGGACTCTG | GCCAGGTTGATCTCAGACTCTTT^a^ |
| **1.1s – 1.2as** | TTAGACAAGGAGAGAAAGATGG^a^ | GTTACTCTCCTCCCTTTCTCG^a^ |
| **1.2s-1as** | ACAGGGAAGAGGGGAAGAGG^a^ | GGGAAGGTAAGTGGGAAATGG |
| **2s-2as** | GATCCCAGCCTTGTACCCAG | GCTTCCGCTGGTGGCTGA |
| **3s-3as** | GACCCTCAGCCACCAGCG | GCACTGAGAAGGACTTGAAGATTG |
| **4s-4as** | CCAGCCTCTCCTCTCCCAG | ATCTTTTCTGGGGCCCTTAG |
| **5s-5as** | CAGGCAGTCCAGAAAGTCGG | ATGAAGAATTGGGTCCCAGATG |
| **6s-6as** | GACTCCCCAAATTTCAGATG | GCCCATCCACTCTTTCCAG |
| **7s-7as** | TCAGGCACTCAGAGAAACATGG | GTCCCCCCATTCCCCATG |
| **8s-8as** | AATGGGGGGACAGTGGGG | TCACAGACCAGCCCAGACAG |
| **9s-9as** | TCCTGTTCTGTCTGGGCTGG | AAGGAGAAAGCCCCCCAG |
| **10s-10as** | CATGACTCGTGGATAGGGAAGG | TGAGGCTTGGGGGCATTG |
| **11s-11as** | AATGCCCCCAAGCCTCAG | CTCATAGCATTTGTGTCTTTCCTG |
| **12s-12as** | TTCCACTCCCCACTGCTTTG | CTGGCTCTGTCCCTCCCG |
| **13s-13as** | GGAGGGACAGAGCCAGGTG | GAGGCTGGAGAGGCACTAGG |
| **14s-14as** | CCTCTCTGGTCTGGCCTCG | TTAGGGTCAAGAAGGCATCG |
| **15s-15as** | CTTAGCTCTGAACTTGTTACCTTG | AGAATAAGGGACCTGGCAGG |
| **16s16as** | AGGGAAGTGGTCATGGGGAG | GGAGACTCCACAATGGGCAAG |
| **17s-17as** | CTAAGACATCCCTCCCACCTG | CATGCCCTGGCGAGTATATAG |
| **18s-18as** | GTAAATGGATAGATGGATGACAGG | GGGTTCAGTGGCAGGTCTTG |
| **19s-19as** | TTACCCAAACACCTGACTTCAAG | TCCTCCACCCATTCGTCTTC |
| **20s-20as** | GTGGATGAAAAGATAAATGGATG | ACTCCATCCTCACACATACACAG |
| **21s-21as** | GCCAGAGCAGTGTTCACCATG | GGACAGGGGGATAGTAAATTCAG |
| **22s-22as** | GAAGGGAATGGGCTAGGG | CTTTTACTAGTTGTGTGACCTTGG |
| **23s-23as** | TTAGCGTTACCATTAGAATTGC | ATCACATGCCTTGGCCTC |
| **24s-24as** | GGCAGAAAGGGTGGGCAC | GGTCTCCACCCTGGCAGG |
| **25s-25as** | CCTGTGGTTGCTGCATACTGAAG | GGCTCTCCTCATATTCGTTCCTG |
| **26s-26as** | GTAAAGAAGGCTCTGAGGGAGG | CCCCACACGCAAAACAAAC |
| **27s-27as** | CACAATCAGGGCACCGACG | AGGCACCCAGTCCAGGCG |
| **28s-28as** | GCCTGGACTGGGTGCCTTG | GTTGGGATTACAGGCATGGACC |
| **29s-29.1as** | GGAGGCGGAGGTTGCAGTG | GGAAACCTCTGCTGTGCTCTCTG^a^ |
| **29.1s-29as** | GAGGAAAAGAATATGTACTGTGTG^a^ | GATCCTCTTACTGGGCTGTTA |

^a^Primers specific for sequencing.

**Methods**

*PCR reactions and sequencing*

PCR reactions for all three genes were carried out for 30 cycles with denaturation at 95˚C for 1 minute, annealing ranging from 53˚C to 65˚C for 1 minute; extension 72˚C from 1 to 2.5 minutes. Denaturing first cycle was carried out for 5 minutes and extending last cycle for 10 minutes. Before sequencing, PCR products were purified using the Wizard® SV Gel and PCR clean-up system (Promega, Madison, WI, USA). Further direct sequencing using ABI PRISM Big Dye Terminator v3.1 Cycle Sequencing Kit (ABI PRISM/PE Biosystems, Foster City, CA, USA) was carried out in two reactions, using sense and antisense primers. When amplicons were longer than 1 kb, internal primers for sequencing were applied. Sequences were obtained in an ABI PRISM 3130 DNA Analyzer (ABI PRISM/PE Biosystems).

**TABLE S4: *NPHS2* and *NPHS1* missenses *in silico* predictions**

| ***NPHS2 missenses*** | **SIFTª** | **PolyPhen^b^** | **Align GVGD^c^** |
| --- | --- | --- | --- |
| p.Arg229Gln | Tolerated  0.11 | Probably damaging  0.903 | Class C35  GV = 0.00; GD = 42.81 |
| p.Val260Glu | Damaging  0.00 | Probably damaging  1.00 | Class C65  GV = 0.00; GD = 121.34 |
| p.Ala284Val | Damaging  0.00 | Probably damaging  1.000 | Class C65  GV = 0.00; GD = 65.28 |
| p.Glu310Lys | Tolerated  0.20 | Probably damaging  0.992 | Class C55  GV = 0.00; GD = 56.87 |
| ***NPHS1 missense*** | **SIFTª** | **PolyPhen^b^** | **Align GVGD^c^** |
| p.Arg408Gln | Tolerated  0.24 | Probably damaging  1.000 | Class C35  GV = 0.00; GD = 42.81 |

^a^SIFT: ≤ 0.05 = damage; > 0.05 = tolerated

^b^PolyPhen: 1.000 = probably damaging; 0.5000 = possibly damaging; 0.000 = benign

^c^Align GVGD: Class 45, class 55 and class 65 = high damaging risk; Class 35 = medium damaging risk; Class 25 = Unclassified; Class 15 and C0 = benign.
